# Impact of six sigma estimated using the Schmidt-Launsbyn vs. the Westgard equation in the Spanish type I EQA program

**DOI:** 10.1515/almed-2025-0091

**Published:** 2025-07-02

**Authors:** Fernando Marqués-García, Elisabeth González-Lao, Xavier Tejedor-Ganduxé, Beatriz Boned, Jorge Díaz-Garzón, Margarida Simón, Jose Vicente García-Lario, Carme Perich, María Pilar Fernández-Fernández, Luisa María Martínez-Sánchez, María Muñoz-Calero, Ricardo González-Tarancón, Pilar Fernández-Calle

**Affiliations:** Department of Clinical Biochemistry, Laboratori Clínic Metropolitana Nord, Germans Trias i Pujol University Hospital, Badalona, Barcelona, Spain; Spanish Society of Laboratory Medicine (SEQC^ML^), Analytical Quality Committee, Barcelona, Spain; Consorci Sanitari de Terrassa, Barcelona, Spain; Department of Clinical Biochemistry, Hospital Royo Villanova, Zaragoza, Spain; Department of Laboratory Medicine, La Paz University Hospital, Madrid, Spain; San Cecilio University Hospital, Granada, Spain; Department of Laboratory Medicine, Central University Hospital of Asturias, Oviedo, Spain; Clinical Biochemistry Group, Vall d’Hebron Research Institute, Vall d’Hebron University Hospital, Barcelona, Spain; Reina Sofía University Hospital, Córdoba, Spain; Department of Clinical Biochemistry, Miguel Servet University Hospital, Zaragoza, Spain

**Keywords:** six sigma, EQA, Wetsgard equation, Schmidt-Launsbyn equation

## Abstract

**Objectives:**

Six sigma methodology (SM) measures process performance using defects per million opportunities (DPMOs). SM has traditionally used the Westgard equation (WM), by which DPMOs are calculated indirectly. An alternative for directly calculate DPMOs is the Z-transformation method in combination with the Schmidt-Launsbyn equation. The implementation of SM in External Quality Assurance (EQA) programs is limited, which hampers their evaluation. A study was conducted to compare SM values obtained with the two equations.

**Materials and methods:**

Sigma value (SV) was estimated based on data from a Type I EQA Program (SCR-EQA-SEQC^ML^) using two methods: the Westgard equation, and the Z-transformation + Schmidt-Launsbyn method (S-LM). A comparison of the SVs obtained with the two methods was performed.

**Results:**

SVs were calculated from 949 values provided by the EQA program. The results indicate that WM underestimates SV, as compared to S-LM, independently of whether outliers were removed (2.9) or not (1.9). This underestimation occurs as a result of treatment bias rather than imprecision.

**Conclusions:**

Unlike MW, S-LM adjusts for bias, thereby preventing negative SVs. S-LM is not as influenced by outliers as MW and yields more robust SV estimates in EQA programs. This guarantees a more precise evaluation of results and classification of method/system performance.

## Introduction

The six sigma methodology (SM) is a structured approach used for assessing process quality in an objective and quantitative way; this is achieved by calculating how often defects are likely to occur, which is expressed as defects per million opportunities (DPMOs). A level of six sigma indicates that, in a process, fewer than 3.4 errors occur per million repetitions [1]. According to the period of time over which the process is assessed, this strategy can be evaluated in the long (maximum quality 4.5 sigma) and short (maximum quality six sigma) term [2]. The short-term sigma value was traditionally calculated as a 1.5-sigma shift from the long-term sigma value [3]. However, Coskun et al. demonstrated that the true long-term sigma value of six sigma is 4.65 sigma (long-term true), not 4.5 sigma [4].

Sigma value is a measure of variability in a process of six standard deviations from the mean value. This means that a process with a 6 Sigma quality level will yield 3.4 values outside the established limit of acceptability (or DPMOs). A 3-Sigma laboratory process has an acceptable minimum quality [3].

The Westgard equation has been the most widely used equation to calculate sigma value (SV) to date [3]. This equation establishes a linear relationship between bias and the analytical goal defined by the total error (TE), and an inverse relationship with imprecision; it also allows for the indirect estimation of DPMOs.

The primary objective of SM is to directly calculate the number of DMPOs in the processes evaluated, which led us to investigate which was the most suitable approach to evaluate SM in laboratory processes [4]. As an alternative, we suggest estimating DMPO directly using Z-transformation as the gold standard method [5] to calculate sigma metrics. To complement calculations, the DMPO value is converted into a sigma value using the Schmidt-Launsbyn equation (S-L) [6], [7].

Currently, the level of implementation of the SM strategy in External Quality Assurance (EQA) Programs is limited [8]. The integration of SM in EQA programs accounts for a step forward in evaluating EQA performance. These programs are essential for the evaluation of analytical performance and provide guidance on the way method and system performance can be enhanced. The ability to assess laboratory performance depends on the EQA program design, as described in five categories [9]. The most highly recommended programs are those that use commutable control materials with values assigned by reference methods/reference materials, and that include the analysis of sample replicates (Category 1). The Spanish Society of Laboratory Medicine (SEQC^ML^) provide a Category 1 EQA program (SCR-EQA-SEQC^ML^) for 17 biological measurands to ensure a more comprehensive assessment of these methods. Finally, more robust SM calculation methods are needed for an appropriate evaluation of the instruments and methods used in EQA programs.

In this study, the SVs obtained with the Westgard equation were compared to those obtained with the Z-transformation method combind with the S-L equation. The primary objective was to identify the method with the highest performance in evaluating SCR-EQA-SEQC^ML^ data.

## Materials and methods

### Materials

This study is based on the results of the SCR-EQA-SEQC^ML^ program. This program uses commutable control material from fresh human serum with a value assigned by reference methods (Category 1) [10], [11].

The control materials used for this study are described in the publication by Ricos et al. [10]. Control material was supplied by Stichting Kwaliteitsbewaking Medische Laboratorium Diagnostiek (SKML) and prepared at the MCA laboratory (Queen Beatriz Hospital, Winterswijk, Netherlands). A total of six vials of control material at different concentrations (six levels) stored at −80 °C were sent to each participant in a single shipment. Each vial was measured in duplicate for six consecutive days. The results obtained are available on the SCR-EQA-SEQC^ML^ website.

Results were included in the study when at least five laboratories participated in the peer group. For all measurands included in the program, means and coefficients of variation (CV, %) were calculated for each method-instrument at each level of concentration. The measurands included in the SCR-EQA-SEQC^ML^ program are shown in Table 1. The Table includes both the performance specification established by the EQA program based on biological variation [12] and the concentration at which the clinical decision level is defined. This study considers the results obtained collectively – not individually – by the laboratories taking part in peer groups in an EQA, which used the same analytical method/instrument and calibrator traceability [13].

**Table 1:** Performance specifications for the total error (TE) established in the SCR-EQA-SEQC^ML^ program and clinical decisión levels for the measurands included in the study.

Biological measurand	Quality assurance program specifications/TE EQA	Clinical decision limit	
α-Amylase	Optimal BV/6.5 %	120	U/L
ALP	Desirable BV/10.4 %	150	U/L
ALT	Optimal BV/9.2 %	60	U/L
AST	Optimal BV/6.1 %	60	U/L
Total bilirubin	Optimal BV/12.3 %	42.7	µmol/L
Calcium	Minimal BV/3.3 %	2.7	mmol/L
CK	Optimal BV/10.3 %	240	U/L
Chloride	Minimal BV/1.9 %	112	mmol/L
Creatinine	Desirable BV/7.8 %	141.4	µmol/L
GGT	Optimal BV/9.1 %	50	U/L
Glucose	Desirable BV/6.2 %	6.6	mmol/L
LDH	Optimal BV/3.4 %	300	U/L
Magnesium	Desirable BV/3.8 %	1	mmol/L
Potasium	Desirable BV/4.9 %	5.8	mmol/L
Total proteins	Minimal BV/5.1 %	60	g/L
Urate	Optimal BV/6.3 %	475.8	µmol/L
Sodium	Minimal BV/1.1 %	135	mmol/L

TE, total error; EQA, external quality assurance; BV, biological variation; AST, aspartate aminotransferase; ALT, alanine aminotransferase; ALP, alkaline phosphatase; GGT, γ-glutamyltransferase; CK, creatine kinase; LDH, lactate dehydrogenase. Optimal, desirable and minimum refers to the level of specification by biological variation.

### Statistical analysis

The SV was calculated using two different strategies: the equation developed by Westgard (MW) [14], [15], and the Z-transformation method followed by the application of the S-L equation (S-LM). Calculations were made for each instrument-method group and for each of the measurands included.

### Westgard strategy for calculating six sigma

Sigma values (SVs) were calculated by the Westgard method (WM) using SCR-EQA-SEQC^ML^ data for imprecision (CV, %) and bias (SE, %). Imprecision for each measurand and control level was calculated as the percentage of data dispersion. Bias was defined as the difference between the mean value at each control concentration and the value obtained with the method of reference. The specification for the total error (TE, %) of the SCR-EQA-SEQC^ML^ program was established as the limit of acceptability of data distribution (Table 1). SV was calculated by implementing the following formula to each peer group at each concentration level:

$$\text{SV}=\left(\text{TE}-\left|\text{SE}\right|\right)/\text{CV}$$

All parameters in the equation are expressed as percentages (%). The SV estimate was converted into DPMOs using conversion charts (https://sixsigmastudyguide.com/process-performance-metrics/).

### Z-transformation and Schmidt-Launsbyn equation for calculating six sigma

SV was calculated through the Z-transformation strategy followed by the application of Schmidt-Launsbyn (S-LM) equation from the SD values obtained for each magnitude, control level and TE established as a specification by the SCR-EQA-SEQC^ML^ program (Table 1). Firstly, the limits to assess compliance with a specification were calculated as follows:

Upper limit=reference value+TE

Lower limit=reference value−TE

The reference value refers to the target value of the control material, as determined by a reference method (type I EQA program). Normal distribution is defined as a mean (µ) of 0 and an imprecision of 1 (σ). To normalize data distribution, the Z-transformation tool was used [16], which indicates the extent to which a result deviates from the optimal target before falling outside the specification limits. Likewise, Z scores were calculated for the two distribution limits obtained for the peer group at each concentration level. The following formulas were applied:

$$\text{Upper z score}=\left(\text{Upper limit-Laboratory reported value}\right)/\text{SD of the peer group.}$$

$$\text{Lower z score}=\left(\text{Lower limit-Laboratory reported value}\right)/\text{SD of the peer group.}$$

The difference between the standard normal distribution of the upper and lower z score defines the area under the curve (AUC) [3] contained within the specifications established. The difference between the AUC defined by the specifications and the calculated AUC accounts for the defects occurring during the process. The value of this area is multiplied by 10^6^ to calculate the number of DPMOs. Finally, the SV is calculated from the DMPO value obtained through the S-L equation [6], [7]:

$$S-L=0.8406+\left(\sqrt{\left(\left(29.37-2.221\times \mathrm{Ln}\left(\text{DMPOs}\right)\right)\right)}\right)$$

Outlier analysis was conducted using the Tukey method [17].

Statistical analysis and plotting were performed using an Excel spreadsheet (Microsoft Corporation^®^, Redmon, Washington, USA).

## Results

**Figure 1:**
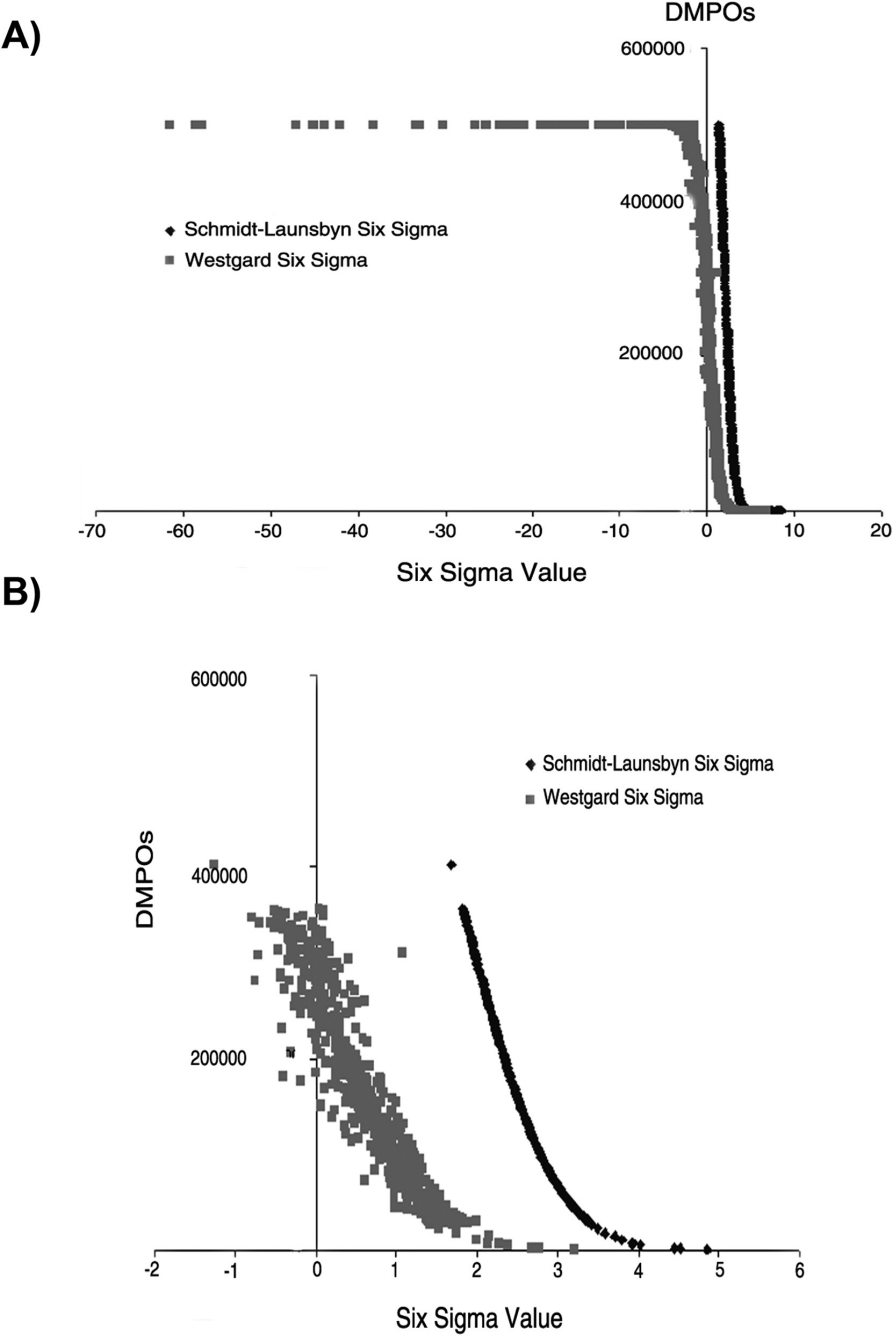
Association between six sigma values and the number of DPMOs according to the MW and S-LM methods. **(**A) Displays six sigma values obtained using the MW and S-LM methods in descending order (from left to right). (B) Provides a detail of the 5 to −1 range of six sigma values from (A). The x-axis represents the six sigma value, while the y-axis shows the number of DPMOs. DPMOs, defects per million opportunities; WM, Westgard method; S-LM, Z-Schmidt-LaunsbyTransformation method. Data for all measurands included in the program are displayed in the Figure.

SM calculations were performed from 949 results (all: concentration levels of the peer group with more than five participants and measurands considered in the program). The SVs obtained were grouped according to the values obtained by the WM and S-LM method. These values were plotted to display the relationship between the number of DPMOs and the SV obtained (Figure 1 and Supplementary Figure 1). Figure 1 shows differences between the SVs estimated by the WM and the S-LM method from a six sigma value to 0. Using the WM equation, sigma values turn negative (up to −60) from 2 × 10^5^ DPMOs (Figure 1A and B). In contrast, negative values were not obtained with the S-LM equation even at DPMO values above 2 × 10^5^. A range from 400 to 1,100 was established, ordering the data from highest to lowest six sigma value (Figure 1B); results were subsequently plotted. In this zone, DPMOs and SV exhibit a unique proportional relationship when the S-LM equation is implemented (Figure 1B).

Conversely, when the WM equation is used, a cloud of dots is observed, showing different SVs for the same number of DMPOs (Figure 1B), and vice versa. Supplementary Figure 1 displays the evolution of the curve plotted in Figure 1A after adding 100 values to each graph. The mean SV for all SCR-EQA-SEQC^ML^ values was obtained from the SVs calculated both by the WM and the S-LM method both including and excluding outliers. When outliers were removed, the mean SV obtained by the WM increased, whereas the mean obtained by S-LM remained unaltered. No statistically significant differences were observed between the two groups when outliers were removed (Table 2).

**Table 2:** Average six sigma values considering the total data of the EQA program at the clinical decision level.

Selected data	S-L	W	(S-L)-W	Imprecision, %	Bias, %
Clinical decision level with outliers	3.20	0.52	2.67	3.49	7.36
Clinical decision level without outliers	3.23	1.33	1.89	3.12	3.90
All EQA data with outliers	3.06	0.31	2.75	3.77	8.33
All EQA data without outliers	3.10	1.14	1.96	3.23	4.37

S-L, Schmidt-Launsbyn; (S-L)-W, Schmidt-Launsbyn method minus the Westgard method; EQA, external quality assurance; W, Westgard**.**

For the mean SV obtained with the S-LM approach, minor differences were observed between groups both when outliers were included or excluded (3.06–3.10) (Table 2). In contrast, when comparison was performed using the data obtained with WM, greater differences, ranging from 0.31 to 1.14, were observed. In the two groups, when outliers were removed, bias decreased, but not imprecision (Table 2 and Figure 1A).

The Schmidt-Launsbyn-Westgard ((S-L)-W) relationship is useful for estimating the difference between the SVs obtained with the two methods. The difference in the SV calculated using the (S-L)-W method ranges from approximately 2.7 to 1.9, depending on whether outliers are included or excluded from the data set, respectively (Table 2). The findings indicate that WM tends to underestimate SV, and that this bias can be mitigated by excluding outliers.

To visualize the tendency of individual SVs, plotting was performed considering all SCR-EQA-SEQC^ML^ measurands at the clinical decision level (Supplementary Figure 2). The S-LM value was considered to sort results in ascending order, proving the proportional difference (underestimation) between the WM SM and the S-LM SM (Supplementary Figure 2). When WM SVs are negative, this difference between the two methods becomes exponential. In contrast, the difference between the two methods remains constant when SVs are positive. Data analysis was performed following outlier removal. When outliers are included, the exponential relationship derived from negative MW SVs intensifies, thereby challenging the visualization of results in the linear zone of the relationship (Supplementary Figure 3).

**Figure 2:**
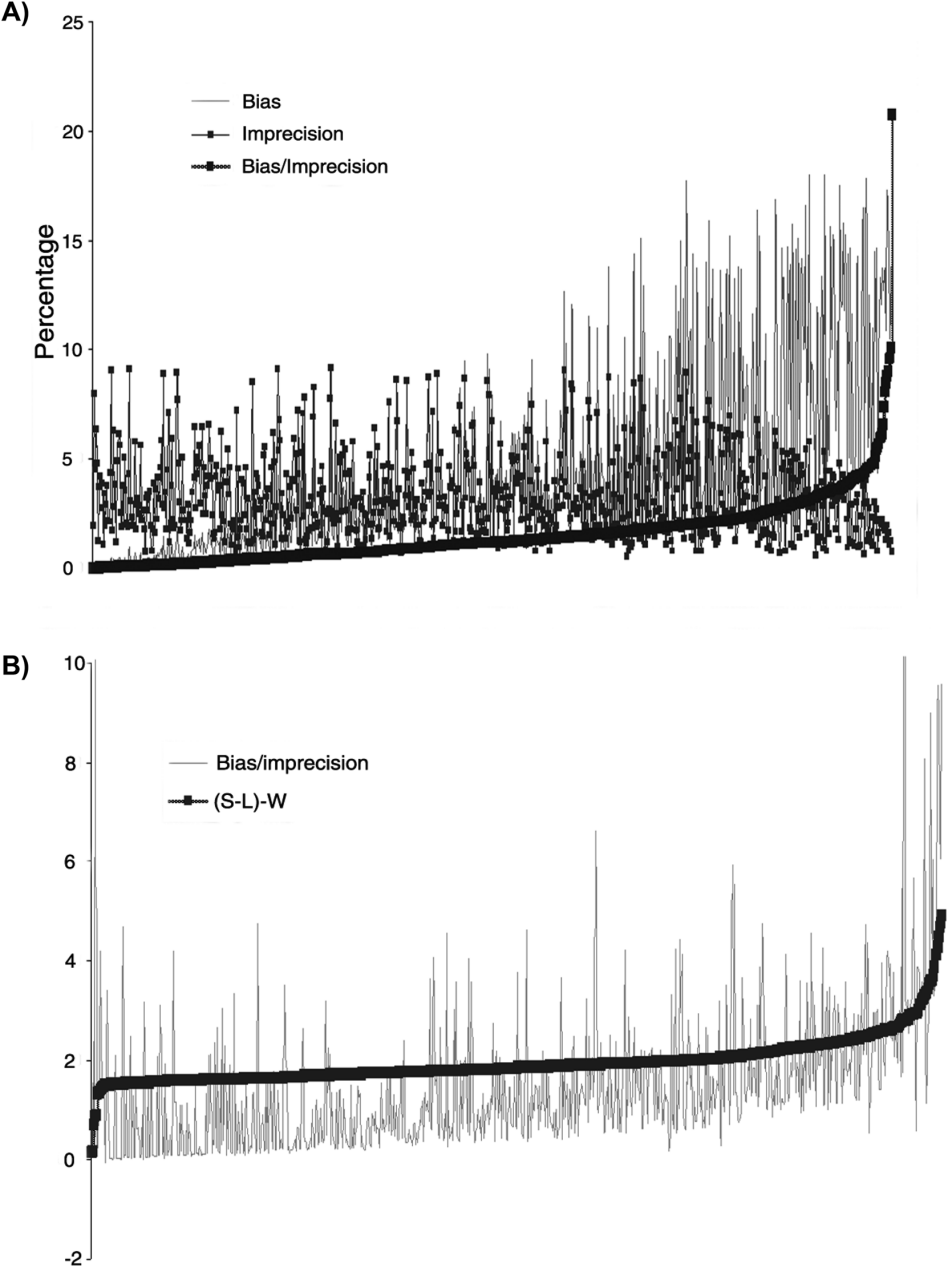
Impact of bias and imprecision in the EQA program on the estimation of the average six sigma value for the two methods. The figures display all EQA data sorted by the bias-to-imprecision ratio (A) and by the difference between the S-LM and MW six sigma values, denoted as (S-L)–W (B), in ascending order. Outlier values have been excluded from both graphs. Data in figures A and B are arranged from left to right in increasing order of the bias/imprecision ratio and (S-L)–W difference, respectively.

Figure 2 displays the relationship between bias and imprecision of the reported results, as obtained from peer groups of laboratories participating in the EQA program, after outlier removal. When these data are ordered from the lowest to the highest bias/imprecision ratio, the increase in the ratio is mainly attributable to a rise in bias. Imprecision remains constant or even slightly decreases at the higher values of the bias/imprecision ratio (Figure 2A). Likewise, an increase in the value of the (S-L)-W difference increases the bias/imprecision ratio, which demonstrates the effect of bias on the WM SV calculation method (Figure 2B). The effect on bias increases when outliers are included (Supplementary Figure 4).

Finally, based on the SVs obtained through the S-LM method, measurands were grouped into three groups: >4 sigma (3 measurands); 4-3 sigma (7 measurands) and <3 sigma (8 measurands) (Table 3).

**Table 3:** Measurands of the SCR-EQA-SEQC^ML^ program grouped by sigma value obtained with the S-LM method.

Sigma value	Measurand
>4	CK
	Potassium
	Enzymatic creatinine
Between 4 and 3	Total proteins
	ALP
	GGT
	Jaffé creatinine
	Urate
	Glucose
	Total bilirubin
<3	Calcium
	ALT
	α-Amylase
	Chloride
	Magnesium
	LDH
	AST
	Sodium

EQA, external quality assurance; AST, aspartate aminotransferase; ALT, alanina aminotransferase; ALP, alkaline phosphatase; GGT, γ-glutamyltransferase; CK, creatine kinase; LDH, lactate dehydrogenase.

## Discussion

### Comparison of the two methods

Based on the literature available, two methods were selected in the Clinical Laboratory to calculate SV. SVs have been traditionally calculated indirectly using the Westgard method (WM) by establishing a proportional relationship between analytical errors: total error (TE), bias and imprecision [18]. Alternatively, in the recent years, some authors have suggested calculating SM based on a direct estimation of DPMOs [19]. This strategy involves transforming data into a normal distribution through a Z-transformation process, which has been described as the most robust method for calculating SM [5]. The relationship between DMPOs and SV is not linear but exponential [20], [21]. In this study, the S-L equation was implemented to transform DMPOs into a sigma value exploiting the logarithmic relationship between these two values (S-LM). This equation yields a single, directly proportional SV for each DPMO value. In contrast, the WM estimates SV indirectly without considering the DMPOs. As a result, different bias/imprecision combinations generate the same SV (Figure 1).

Additionally, as analytical bias increases, aberrant (negative) SV values tend to appear from approximately 2 × 10^5^ DMPOs. When bias exceeds the established distribution limits (e.g. the quality specification for TE set by the EQA program) the aforementioned negative values begin to appear. This occurs due to the linear treatment of errors (primarily bias) assumed in the Westgard equation [3], whereas the relationship between SV and DMPOs follows a normal distribution [22]. No variations were noted in the behaviors described when all data are considered or when analysis is focused on values close to the clinical decision level. In the light of the problems that calculating SV by the Westgard equation entails, outliers were removed from data distribution (the highest bias values), resulting in a significant – but not total – reduction of negative SVs. On another note, no variations were observed in imprecision according to the method used to calculate SM (Westgard vs. S-L) or the treatment of outliers (presence vs. absence).

Likewise, the average SV for the SCR-EQA-SEQC^ML^ as calculated by the S-LM method did not vary significantly when outliers were maintained or removed, either considering the entire data set (3.06 vs. 3.10) or solely clinical decision levels (3.20 vs. 3.23). However, variation in the WM SV increased as a function of whether outliers were removed or not, both at the clinical decision level (0.52 vs. 1.33) or when all SCR-EQA-SEQC^ML^ program data were considered (0.31 vs. 1.14). The bias/imprecision ratio decreased after outliers were removed due to a reduction in bias. Therefore, the SVs obtained by the S-LM method are not influenced by outliers, in contrast to those calculated with the Westgard method, which are influenced by bias. The way bias is approached by each method definitely contributed to the differences observed in SV. The difference between the SVs obtained with the S-LM and the WM method is constant. Hence, WM underestimates the SV, ranging from 2.7 when outliers are included to 1.9 following outlier removal. Differences in SV between the two strategies ((S-L)-W) increase as bias increases (elevated bias/imprecision ratio) (Figure 2A and B).

Underestimation of the sigma value by the Westgard method hinders the achievement of the SCR-EQA-SEQC^ML^ quality objectives. The WM is significantly influenced by high bias values, thereby resulting in negative SVs. This problem is overcome with the S-LM method, which treats bias value more appropriately. Proper bias management contributes to an enhanced assessment of the performance of methods and instruments, as well as of the EQA program as a whole.

### Performance of the EQA program

EQA programs are aimed at assessing analytical performance. The WM approach yields inappropriate sigma values ranging from 1.33 when outliers are considered to 1.14 when removed, both at the clinical decision level. In contrast, the S-LM strategy yields values ranging from 3.23 to 3.10, respectively. This way, the overall performance of the program would exceed the SV limit accepted in the clinical laboratory, established at three sigma. This sigma value provides a general picture of the EQA program performance. The availability of a sigma value for each measurand provides guidance as to the measures to be adopted to improve analytical performance, where appropriate. Only three measurands exceeded four sigma (CK, potasium and enzymatic creatinine); seven measurands received a 4-3 sigma; and eight had a <3 sigma. In view that three sigma is the lowest value, clinical laboratories and/or the *in vitro* diagnostics (IVD) industry should intensify efforts to improve analytical performance in measurands with a SV<3. Moreover, performance should be enhanced in measurands with a 4-3 sigma to reach sigma 4. Creatinine deserves special attention, as the most precise method –enzymatic creatinine– exhibited a better sigma value (SV>4) as compared to the Jaffé method (SV between 4 and 3). This is a clear example of how clinical laboratories should use the analytical methods with the best performance. The SM calculation method has a significant impact on the SV obtained and affects the fulfilment of EQA program objectives.

The six sigma metrics emerges as a valuable tool in EQA programs, as it provides a picture of the analytical performance (imprecision and bias) of laboratory methods and instruments through a single indicator. In addition, SM facilitates the harmonization of different EQA program, providing a benchmarking parameter.

Currently, the level of implementation of six sigma metrics within EQA programs is limited. The Dutch program SKML provides SV in the performance assessment report, but uses the Westgard method, which has important limitations, as demonstrated in this study. Table 3 displays the SCR-EQA-SEQC^ML^ measurands grouped according to their six sigma value. The assessment of analytical performance presented in this table is cumulative for each measurand. Thus, each EQA program must be evaluated separately for each instrument and measurand. However, this is out of the scope of our study and will be addressed in future studies. In general, we encourage the IVD industry to enhance analytical methods for measurands with a low sigma value to achieve the analytical performance established based on biological variation. For instance, in the case of sodium, direct potentiometry should be used instead of indirect potentiometry to improve its six sigma value.

In conclusion, the S-LM method complies with the requirements for a correct calculation of SM, including ensuring the normal distribution of the data set. In addition, it overcomes the limitations of the WM methodology. Thus, the S-LM method calculates DMPOs directly and enables subsequent estimation of the SV, in contrast to the Westgard equation. With the S-LM, each DPMO value corresponds to a single SV. S-LM yields more robust SVs, as they are not influenced by outliers. Unlike WM, the S-LM method involves a non-linear treatment of bias. This approach reduces the impact of bias on SV. The results of this study underline the significant impact of bias on SV and the relevance of selecting the most appropriate strategy for calculate SM. A robust SM calculation method will pave the way to its inclusion in EQA programs, thereby resulting in a more objective assessment of analytical performance.

## Supplementary Material

Supplementary Material
