# Supplementary material for: Impact of six sigma estimated using the Schmidt-Launsbyn vs. the Westgard equation in the Spanish type I EQA program
Source: Adv Lab Med. 2025 Jul 2;6(3):327–35. doi: 10.1515/almed-2025-0091 (PMC12446911; doi:10.1515/almed-2025-0091)

**Supplementary Figure 1.** Evolution of the Six Sigma curves calculated using the two proposed equations as EQA data are added 100 by 100.
These graphs were generated by sorting the EQA data according to the number of Defects Per Million Opportunities (DPMOs).


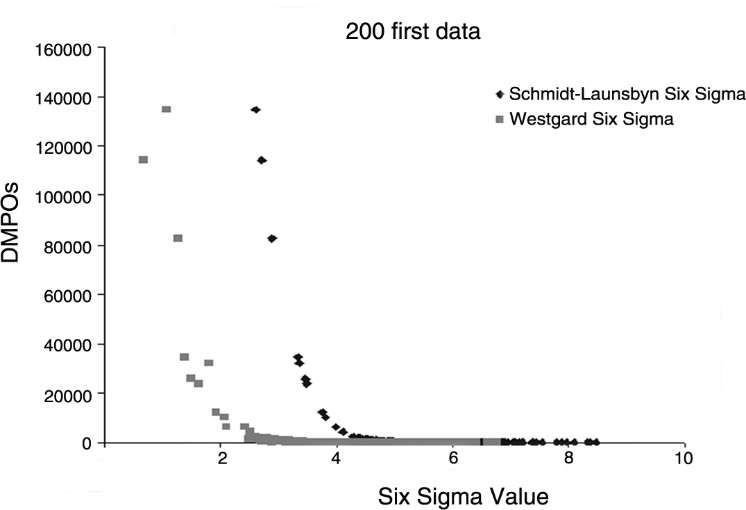

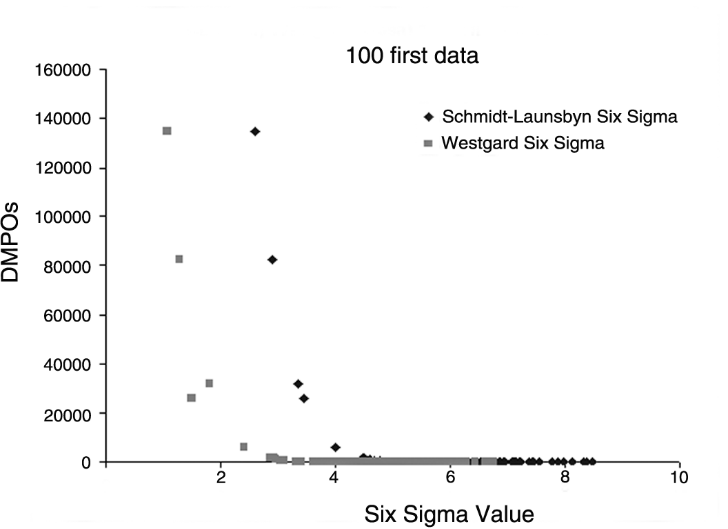


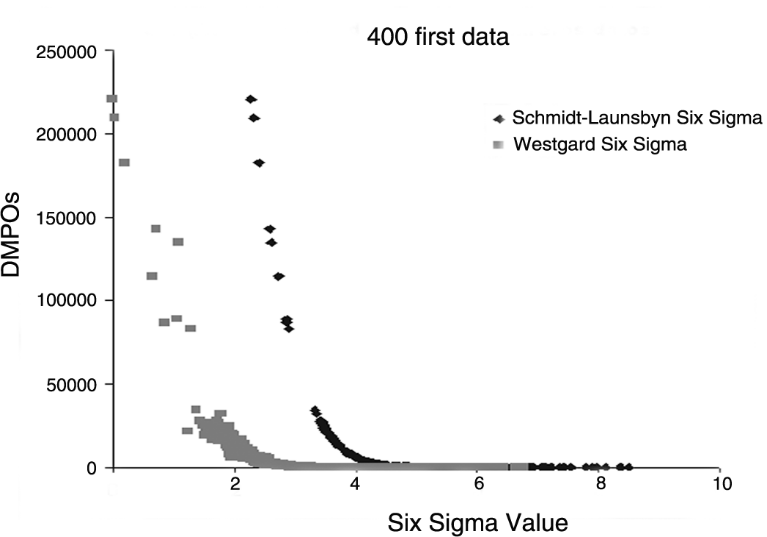

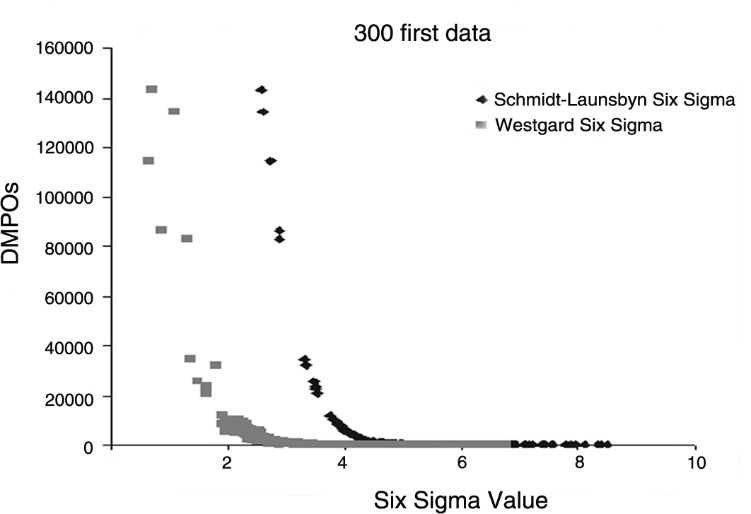


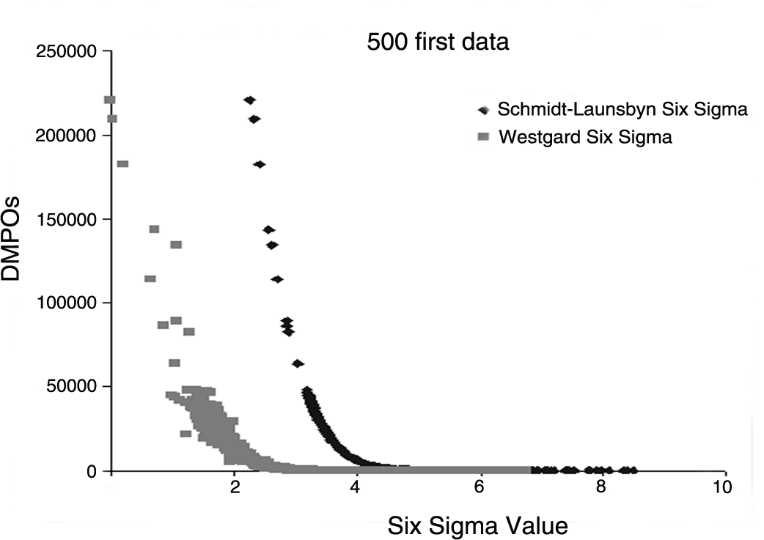


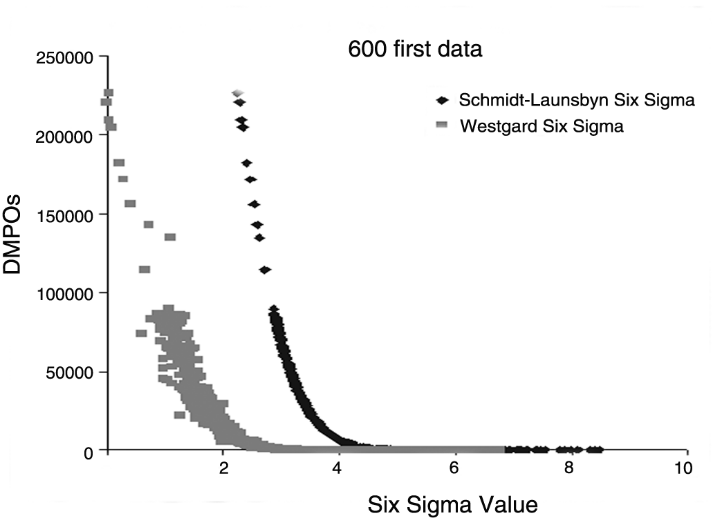


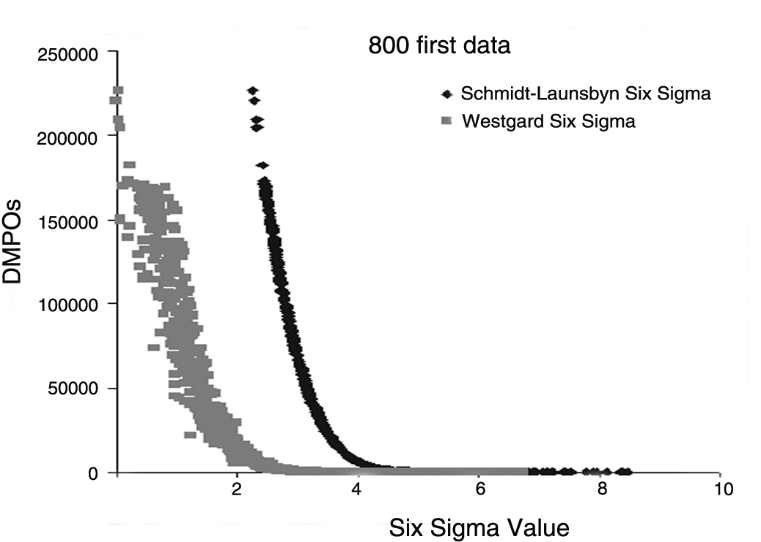


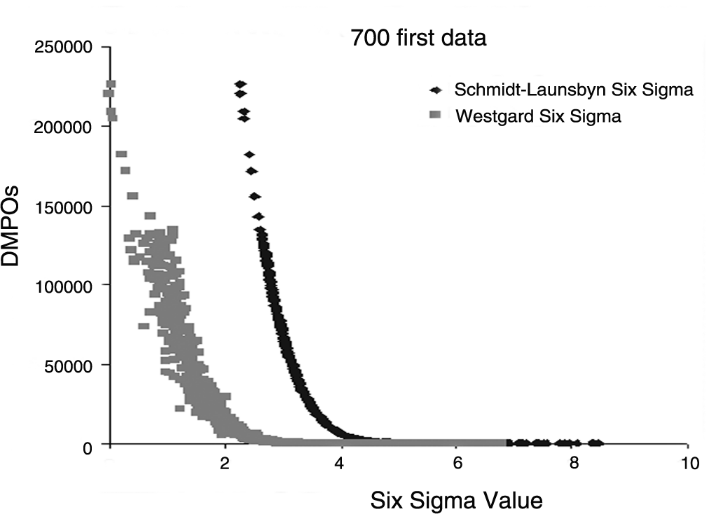


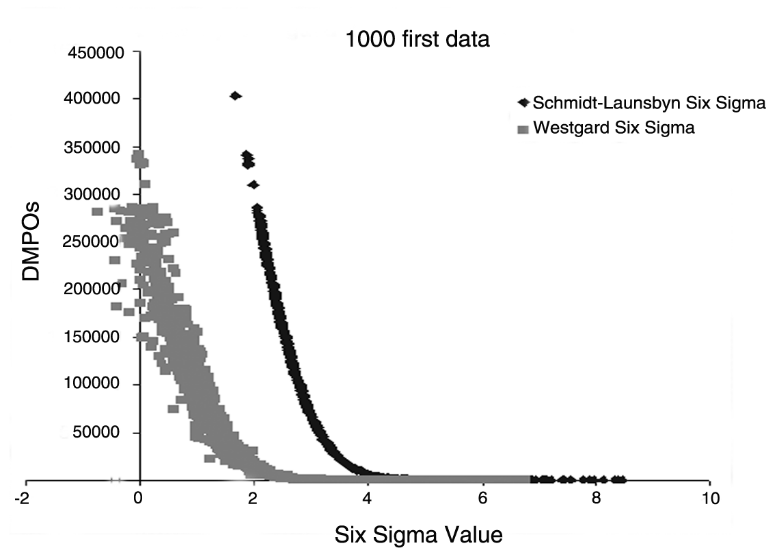

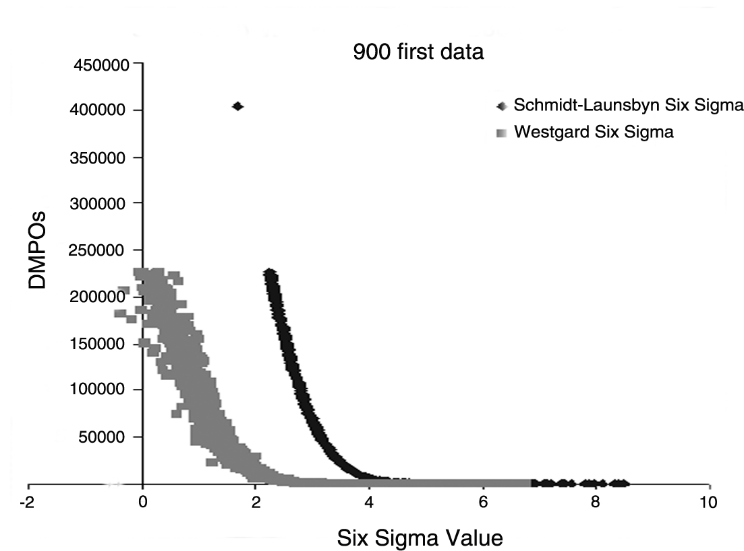


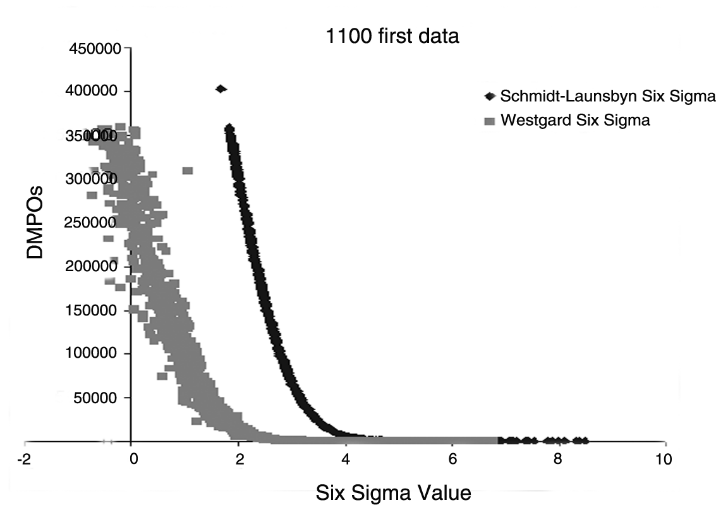

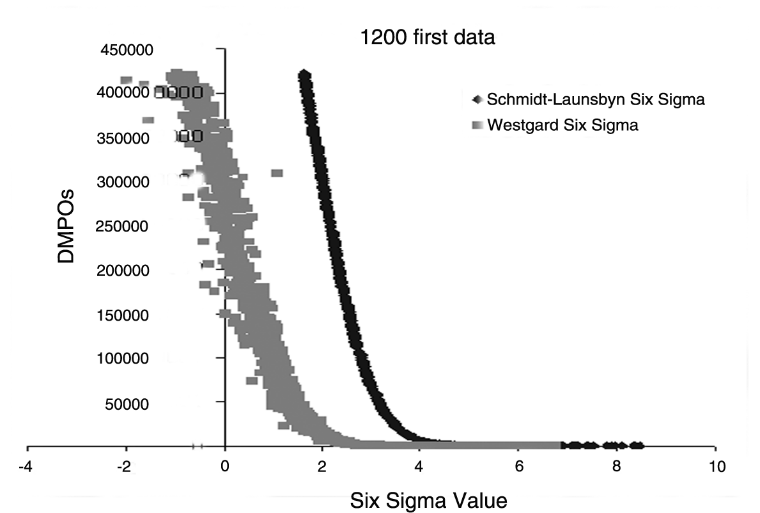


**Supplementary Figure 2**. Relationship between the sigma values calculated by the Westgard method (W) and the Z-transformation–Schmidt-Launsby equation (S-L).
(S-L)-W: accounts for the difference between the six sigma values obtained by the S-L and the W method. Data are sorted in ascending order of S-L values, following the removal of outlier values, at the clinical decision limit.


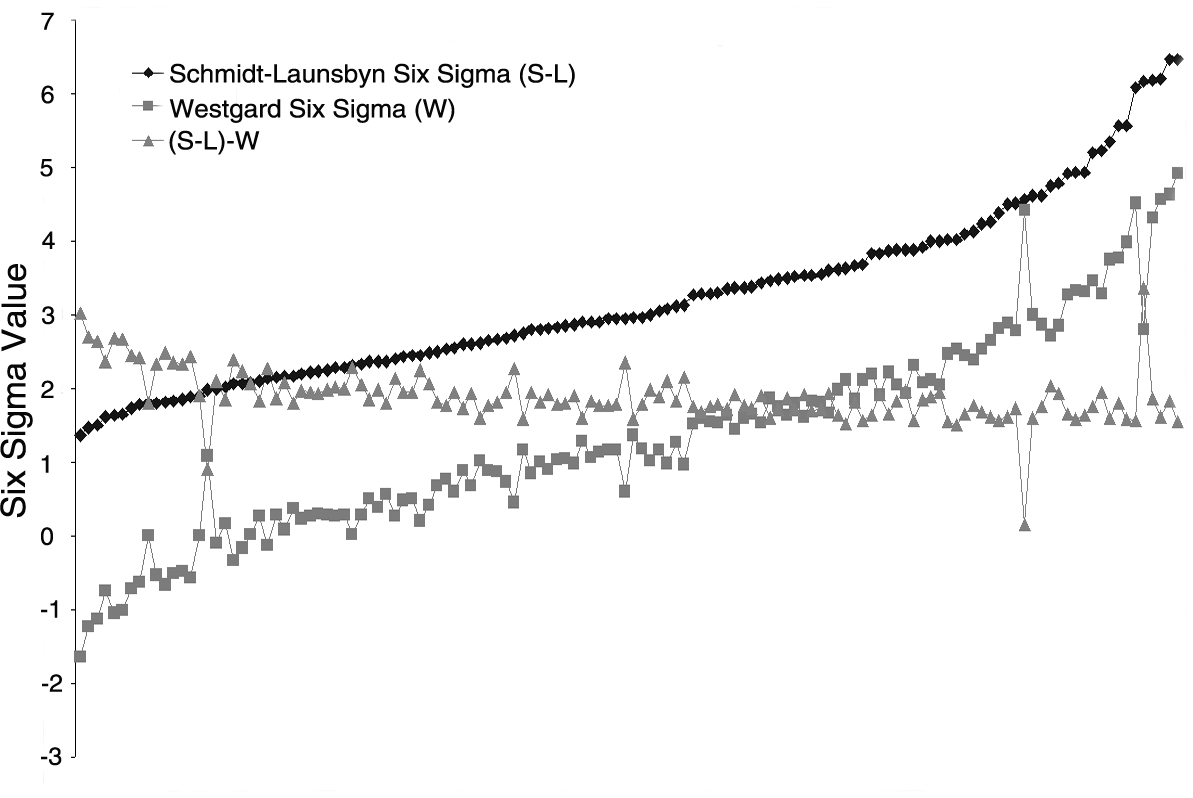


**Supplementary Figure 3.** Relationship between the sigma value calculated using the Westgard method (W) and the Z-transformation–Schmidt-Launsby equation (S-L).
(S-L)-W: denotes the difference between the six sigma values obtained by the S-L and the W methods. Data are sorted in ascending order of S-L values, including outlier values, at the clinical decision threshold.


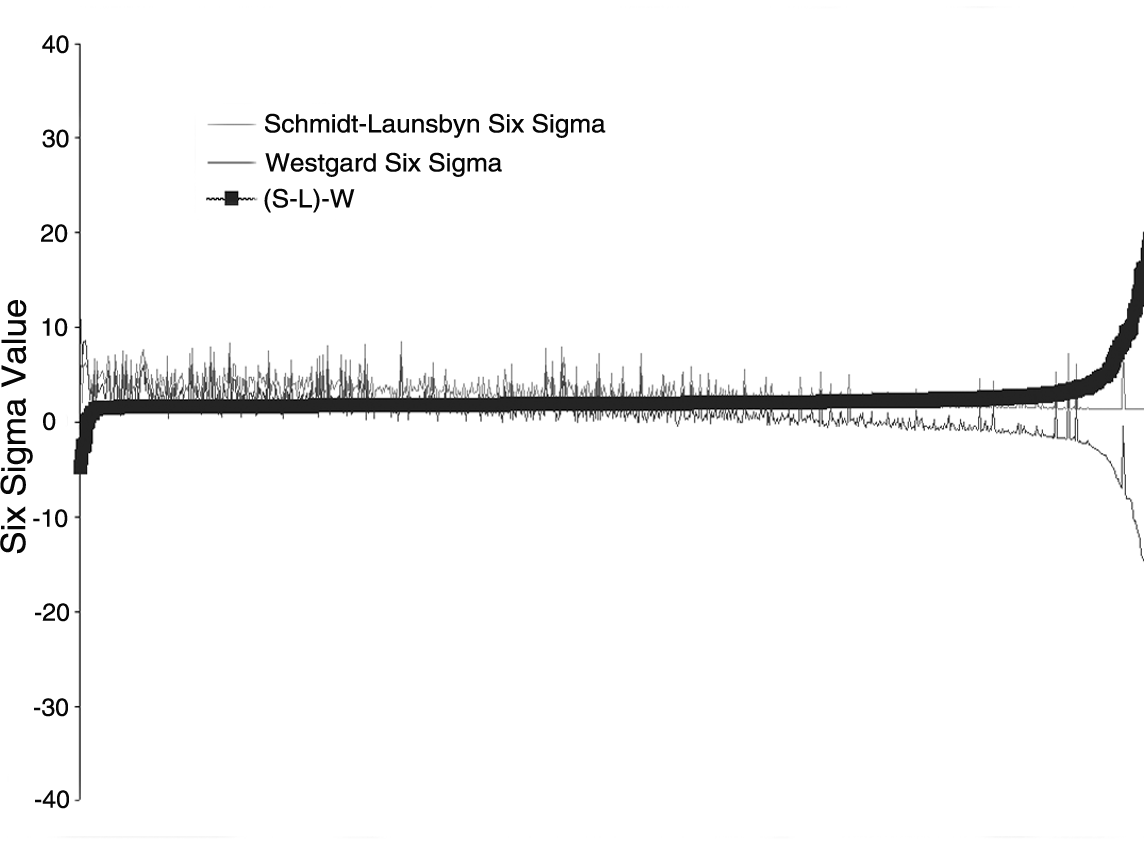


**Supplementary Figure 4.** Relationship between the bias percentage, imprecision and the bias/imprecisión ratio. Outlier values are included.


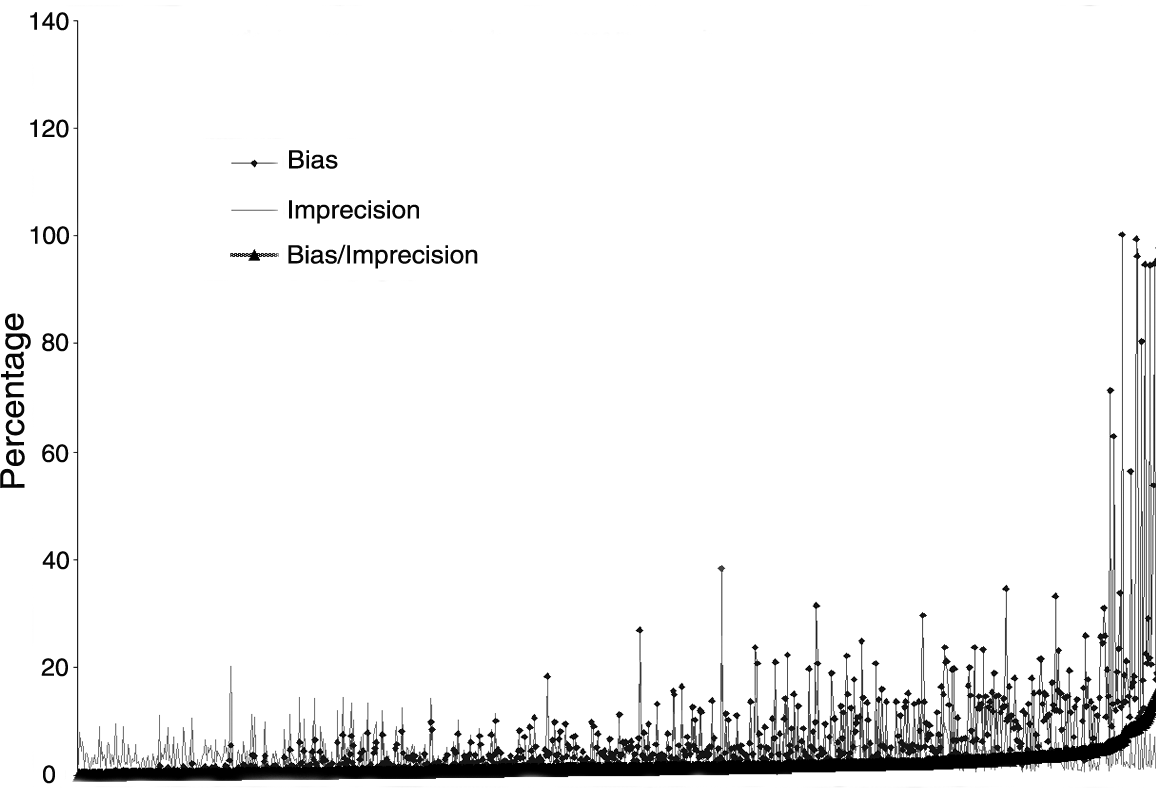

Supplement: Supplementary file 1 — Supplementary Material [file j_almed-2025-0091_suppl_001.docx]
